# Achieving equity for International Medical Graduates: a systematic review

**DOI:** 10.3389/fmed.2025.1601492

**Published:** 2025-07-23

**Authors:** Sangeeta G. Saxena, Elizabeth Tisdell, Elana Farace, Thomas Godfrey, Betsy Aumiller, Esther Dell, Omrana P. Razzak, Bernadette N. Kumar, Kristin K. Sznajder

**Affiliations:** ^1^Department of Public Health, Coastal Carolina University, Conway, SC, United States; ^2^Penn State Harrisburg, Middletown, PA, United States; ^3^Penn State Milton S. Hershey Medical Center, Hershey, PA, United States; ^4^City College of New York (CUNY), New York, NY, United States; ^5^Norwegian Institute of Public Health (NIPH), Oslo, Norway

**Keywords:** IMGs, acculturation, training, high income countries, equity

## Abstract

**Introduction:**

Foreign-born and foreign trained International Medical Graduates (FIMGs) face greater challenges in acculturation to their host countries than IMGs who train abroad and return to practice in their home country. As FIMGs are likely to fulfill a shortage of physicians in High Income Countries in the foreseeable future, we conducted a systematic review of literature to identify acculturation interventions that help FIMGs assimilate better in their host country health systems. This improves their productivity and satisfaction, allows health systems to be more accepting of FIMGs, and most importantly, enhances patient outcomes.

**Methods:**

Following the PRISMA statement, we searched PubMed, Embase, PsycINFO, CINAHL, Web of Science for all peer-reviewed articles using keywords “international medical graduate”, “overseas trained doctor”, “overseas trained physician”, “foreign trained doctor”, “foreign trained physician” (group A); and “discrimination” and “microaggressions” (group B) published between January 1st, 2000 to October 24th, 2021.

**Results:**

The 46 studies included in this review fall into three groups – acculturation interventions for FIMGs, FIMG’s perceptions of what they found useful, and trainers’ perspectives on ‘what works’. This review also includes interventions that pivoted to the online mode during the Covid-19 pandemic, making the findings relevant, as this is likely to the norm in the future. Acculturation requires training on clinical protocols, host country and health system culture and norms and communication, language and self-awareness skills.

**Discussion:**

Much work remains to be done. Interventions need to be tailored to suit the unique needs of FIMGs from 150+ countries, trainings require a foundation of theoretical frameworks, additional professional, personal and social support to be provided, life course related changing needs demand attention and the preparedness of host country health systems to accept FIMGs require enhancement.

## 1 Introduction

International Medical Graduates (IMGs) are physicians who graduate with their primary medical degree from a country that is not their host country. They fulfill the physician shortage the HICs face, and this trend is projected to continue in the foreseeable future (1). A distinct subset of these IMGs are physicians for whom both, their country of origin and primary medical training is not their host country, and these IMGs constitute a significant percentage of the physician workforce in High Income Countries (HIC)^1.^ This review focusses on this subset of IMGs for their transition to practice in their host country health systems is fraught with more challenges than for IMGs who train abroad and return to their country of origin. Hence host country citizens who move abroad for medical training and then return to practice medicine have intentionally been excluded from this review, for they do not face the social, emotional and acculturation challenges that IMGs whose country of origin is not the host country, experience.

Obviating these challenges foreign born IMGs face requires efforts on many fronts. The most evident of these is the need to fulfill educational and legal requirements such as licensing and certification, recognition of credentials and residency matching. A systematic review of the educational interventions supporting IMGs (1) to surmount these challenges exists. However, to utilize the full potential of IMGs, enhance their work-life satisfaction and simultaneously ensure that patients and communities are fully accepting of them requires health systems to work holistically on a number of additional fronts. IMGs may need to enhance their language proficiency, deal with the mental and emotional strain of transitioning to a new country while facing potential isolation from family and home culture, and possibly face bias and discrimination, all the while building a professional and social network. Host communities and health systems may need to prepare to accept IMGs through strengthening their cultural competency and work on ways to culturally and socially integrate them.

Hence, we synthesized evidence on this broader premise that equity and a sense of belonging is the foundation for immigrant physicians to feel “at home” in their host countries, thereby being in a better position to be equal to their peers, enhance their professional lives and contribute to improved patient outcomes.

This evidence of best practices ameliorating IMGs acculturation to life in their host countries can inform and guide future practice about holistically integrating IMGs in host country health systems in the HICs, including the US, which has the projected highest need for immigrant physicians (2). Our aim is in conducting this review is to encapsulate the evidence generated over the past two decades so as to.

## 2 Methods

### 2.1 Study design

This study is a systematic review adhering to the Preferred Reporting Items for Systematic Reviews and Meta-Analyses (PRISMA).

### 2.2 Eligibility criteria

The inclusion criteria are:

1. Studies providing an evidence base of interventions within the scope of the health system aimed at enhancing acculturation for IMGs.
2. Quality improvement projects, Randomized controlled trials (RCTs), case–control studies, cohort studies (prospective or retrospective), cross-sectional studies, qualitative studies, expert commentaries and opinion papers.
3. Published in English between January 2000 to December 2021. The pace of change in society, health systems and medicine has accelerated since the turn of the century, and the wealth of new evidence available on this topic has increased manifold over the past two and a half decades, hence this review was restricted to research done since the year 2000.

The exclusion criteria included the following:

1. Conference abstracts, Conference proceedings.
2. Review articles.
3. Articles not in English.

### 2.3 Search strategy

This review adheres to the revised reporting guidelines and criteria set in Preferred Reporting Items for Systematic Reviews (PRISMA) (3). The search strategy was developed with expert input from a librarian (ED) and no filters for study design were used.

The search was conducted between June 30, 2021, and October 24, 2021 and covered five electronic databases—PubMed, Embase, PsycINFO, CINAHL and Web of Science and Google Scholar was searched for gray literature. No filters for study design were used.

The search was conducted using the different and truncated designations for International Medical Graduate. The phrases used were “international medical graduate,” “overseas trained doctor,” “overseas trained physician,” “foreign trained doctor,” “foreign trained physician” (group A); discrimination, microaggressions (group B) and coping, transition, health system, facilitatory (group C). The MeSH terms/keywords were “Foreign Medical Graduates” (group A), “social discrimination,” “bias,” “implicit bias,” “racism,” “prejudice”(group B) and “acculturation,” “cultural diversity”(group C). These were used with the Boolean operators OR and AND. Although search was planned to sequentially combine the terms in groups A, B and C, the search was stopped after using the search terms in groups A and B because the number of articles found after inclusion of words from group C was small (< 20). Sieving through such few articles could have precluded relevant articles from the findings. Hence larger numbers of results listed from searches of terms under groups A and B were combed to ensure no study fitting the inclusion criteria was missed. A sample of the search conducted of PubMed is available at Appendix.

**Figure 1:**
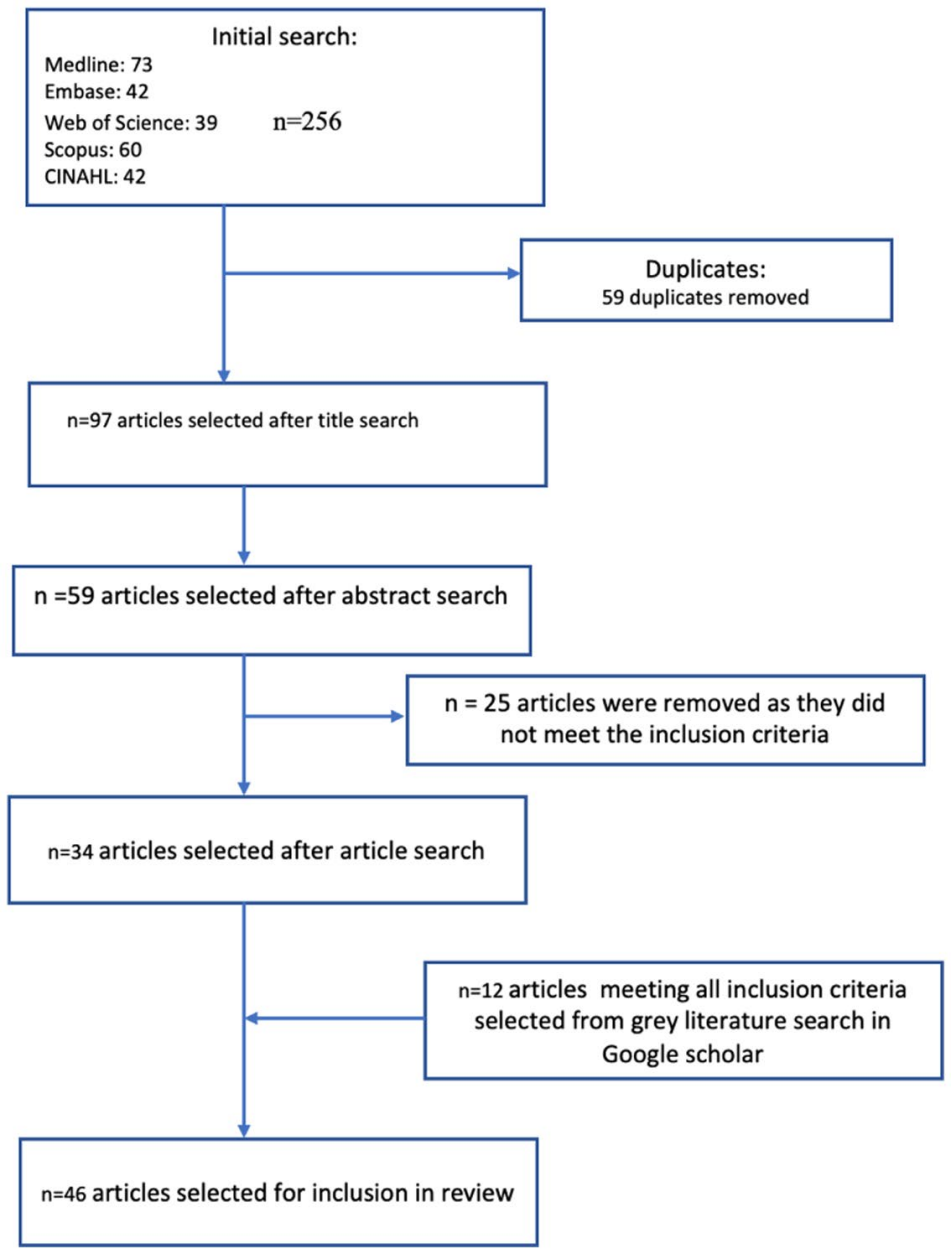
Flow chart of articles selected for and excluded from review.

The screening and selection process is presented at Figure 1. Two researchers (SGS and ED) conducted the search independently. Both researchers resolved differences of opinion regarding eligibility by consensus discussion. A third researcher had been identified (TG) at the start of the review to step in to help resolve any differences of opinion between the two researchers, but this was not needed. Effort was made to contact authors where more details were required. Some authors provided additional information (4–6).

The selected literature is empirical evidence intended for facilitation of acculturation of IMGs in their host countries with the aim of facilitating the navigation of their professional careers. The Population, Issues, Context, Outcomes (PICO) (7) framework was used to create the review protocol (Table 1).

**Table 1:** The Population, Issues, Context, Outcomes (PICO) study design of the systematic review (7).

Population	International Medical Graduates (IMGs) working in High Income Countries (HICs)
Issues	Challenges faced by the above population in their host country
Context	Working in the health sector
Outcomes	Initiatives to address challenges and facilitate acculturation

### 2.4 Assessment of methodological quality

The standardized critical appraisal instruments from the JBI System for the Unified Management, Assessment and Review of Information’s (JBI SUMARI) Critical Appraisal Checklist for Qualitative Research was used to assess methodological quality. Studies that got scores against the appraisal criteria of 7–10 were of high quality; moderate quality if they scored between 4 and 6 and low methodological quality if they scored lower than four.

### 2.5 Data extraction

The JBI standardized data extraction tool—JBI Qualitative Assessment and Review Instrument Data Extraction Tool for Qualitative research, was used to extract data from the included primary studies. The data extracted included details on type of study/paper, participant demographics; study methodology and methods; and findings relevant to the review question. For qualitative studies, relevant quotes have been included. In line with meta-aggregative approach, when extracting findings from the intervention studies, the reviewers allocated a level of credibility to each finding based on the degree of support each intervention had (satisfaction by trainees, perceived usefulness of intervention, objective test of learning included in intervention and measure of on-the-job performance).

## 3 Findings

The studies that were included in this review are primarily of three broad types:

1. Studies documenting acculturation interventions for IMGs,
2. Studies documenting IMGs’ perspectives of what they found useful, and
3. Expert opinion and commentaries of “what works.”

These groups are not mutually exclusive, for some studies included in group I also additionally document IMGs’ and/or their trainers’ satisfaction. Table 2 summarizes the key characteristics of the 46 studies included in this review. The UK leads the quantum of research in all three types of studies identified (18/46). The content of the intervention/support measure provided/recommended in each of the three groups of studies is at Table 3. None of the studies included in this review used comparison groups to assess the effectiveness of their curricula and duration and mode of training.

**Table 2:** Types of studies and the host countries for the studies in this review.

Country	Case/evaluation studies (*n* = 15)	Studies with IMG perspectives on what they perceived useful (*n* = 21)^*^	IMG trainer perceptions (*n* = 10)	Number of countries (*n* = 46)
USA	3	2	4	9
UK	8	4	5	17
Canada	2	4^*^		6
Australia	2	7	1	10
Finland		1		1
Netherlands		1		1
Ireland		1		1
Sweden		1^*^		0
New Zealand		1		1

^*^Neiterman et al. (38), compares Canada and Sweden.

**Table 3:** Course content of trainings.

Study and year	Clinical training	Host country health system, culture and customs	Communication and language skills including active listening and shared decision-making	Self-awareness skills, including feedback skills, clear setting of expectations
Intervention studies with a training component				
Baker and Robson, 2012 (19)	✓		✓	
Bansal et al., 2015 (10)	✓	✓	✓	
Bogle et al., 2020 (9)	✓	✓	✓	✓
Cross and Smalldridge, 2011 (12)	✓		✓	
Fry and Mumford, 2011 (13)	✓	✓	✓	
Fournier et al., 2020 (11)	“Doc-to-doc” psychosocial support and practical/professional guidance over e-mail			
Katz et al., 2020 (14)	✓	✓	✓	
Kehoe et al., 2019 (8)	✓	✓	✓	✓
Makker et al., 2020 (6)	✓		✓	✓
Myers, 2004 (20)		✓		
Pillai and Tran, 2019 (15)	✓			
Porter et al., 2008 (16)		✓		
Sockalingam et al.,2015 (17)	✓			
Whyche, 2009 (18)	✓	✓	✓	
Wright et al., 2012 (21)	✓	✓	✓	
Studies focusing on perceptions of IMGs regarding “what works in training				
Curran et al., 2008 (28)	✓	✓		
Hawken, 2005 (30)			✓	
Hepponemi, 2018 (46)			✓	✓
Huijskens et al., 2010 (31)			✓	
Lockyer et al., 2007 (32)	✓	✓		
McGrath et al., 2009 (35)			✓	
McGrath et al., 2012 (36)				✓
Odeunmi et al., 2021 (26)		✓	✓	✓
Wearne et al., 2019 (43)	✓	✓	✓	✓
Commentaries/viewpoints/perspectives of trainers of IMGs on “what works” in training				
Broquet and Punwani, 2014 (55)				✓
Rao and Roberts, 2020 (5)	✓	✓	✓	✓
Woodward-Kron, Fraser, Pill and Flyn, 2014 (47)			✓	
Zaidi, Dewan and Norcini, 2020 (51)	✓	✓	✓	✓

### 3.1 Group I: studies documenting acculturation interventions

Fifteen studies (6, 8–21) included in this review provide evidence of transition to residency (TTR) orientation programs that have been conducted and assessed over time periods ranging from 6 months to 3 years. In 10 of these programs, the interventions were delivered in a full-time training format (6, 8, 9, 12–18) and in the other five, the intervention was delivered in short, modular sessions spread out over 6 months (10, 11, 19–21). The multifaceted interventions utilized combinations of pedagogical methods including discussions, presentations, videos, role-plays, simulations, language training, mentoring, clinical scenarios, focus group discussions and clinical supervisions. One Canada based case study researches the utility of peer mentoring, conducted in pairs of a volunteer senior IMG and a new IMG, over a period of two years (11).

Strong, objective evidence for the effectiveness of the acculturation interventions is recent. The assessment of the strength of evidence of effectiveness of the intervention has been done by using the GRADE-CERQual (“Confidence in the Evidence from Reviews of Qualitative research”) tool (22), with the strength of evidence increasing from type I to type IV. The results are at Table 4. Of the ten (8, 9, 12, 14–19, 21) studies generating type III evidence, eight (8, 9, 12, 14, 15, 17, 19, 21)have been conducted since 2010 and all four (8, 14, 19, 21) studies with results incorporating type IV measures have been conducted after 2010. Only four studies in this group (9, 10, 13, 14) provide objective evidence of IMGs’ acculturation as well as record the IMGs’ and their trainers’, perceptions of effectiveness. Ten of these studies were one-off courses (6, 12–20).

**Table 4:** Strength of evidence for effectiveness of the training/intervention done in case studies.

Measure	Number of studies	Study and year
I = satisfaction with intervention	11 (6, 8, 10, 13–19, 21)	Baker and Robson, 2012 (19); Bansal et al, 2015 (10); Fry and Mumford, 2011 (13), Katz et al, 2020 (14); Kehoe et al., 2019 (8); Makker et al. 2020 (6), Pillai and Tran, 2019 (15); Porter et al., 2008 (16); Sockalingam et al,2015 (17); Whyche, 2009 (18); Wright et al., 2012 (21)
II = perceived usefulness of the intervention	10 (8, 11–14, 16–21)	Baker and Robson, 2012 (19); Cross and Smalldridge, 2011 (12); Fournier and Tourian 2020 (11), Fry and Mumford, 2011 (13); Katz et al, 2020 (14); Kehoe et al., 2019 (8); Myers, 2004 (20); Porter et al., 2008 (16); Sockalingam et al, 2015 (17); Whyche, 2009 (18); Wright et al., 2012 (21)
III = objective test of learning or behavior	8 (8, 9, 12, 14–19, 21)	Porter et al., 2008 (16); Cross and Smalldridge, 2011 (12); Baker and Robinson, 2012 (19); Kehoe et al., 2019 (8); Pillai and Tran, 2019 (15); Bogle et al, 2020 (9); Katz et al, 2020 (14); Sockalingam et al,2015 (17); Whyche, 2009 (18); Wright et al., 2012 (21)
IV = measure of on-the-job performance	4 (8, 14, 19, 21)	Baker and Robinson, 2012 (19); Wright et al., 2012 (21); Kehoe et al., 2019 (8); Katz et al, 2020 (14)

Among the HICs, UK provides a wealth of evidence. There is documentation about the issues clinicians training IMGs must consider, and how efforts to address these issues have been initiated (23). Two studies (6, 9) describe trainers’ experiences of training programs conducted for IMGs in the online mode during the Covid-19 pandemic and they measure satisfaction of the trainers and the trainees with the intervention. Two acculturation interventions, conducted by the Epsom and the Kings Overseas Doctors Development Programs, have had independent third-party mixed-methods evaluations (8, 9) and another has been evaluated by the organizers (21). These evaluations demonstrate the need for varied components in induction trainings for IMGs and how nimble transition to online channels in times of crisis can be done effectively (9). Another two studies have served as pilots and their findings have been inputs to create systemic courses for IMGs (6, 10) (personal communication). The evaluation measures used range from all four types of evaluation measures (strong evidence) (8) to weaker evidence (type I evidence) in the online acculturation trainings (6, 9). Based on the accumulated evidence, the content of induction trainings has been expanded from clinical training to include information about host country health system, culture and customs, communication and language skills and self-awareness skills. Communication experts have been brought in for training of IMGs on language issues and techniques of communicating effectively with their patients and health system personnel. Delivery of acculturation interventions is now systemic at scale for IMGs in the UK (24).

Another innovation is the use of varied channels of communication, such as the internet, and different software to facilitate the expanded reach and enhance the flexibility of timing of training, all without a concomitant increase in costs. This allows sharing of information before the IMGs leave their home countries and has allowed IMGs to be supported during the Covid-19 pandemic, while enhancing the efficiency of scarce resources.

Only one study in this review provides robust evidence of acculturation training leading to enhanced patient-provider relationships, which in turn, improve patient outcomes (25). This study comprehensively documents the short- and long-term effect of induction trainings on IMGs, its effect on their relationship with colleagues, and reports on feedback from trainers and patients (14). It is a three-year pilot by IMG trainers, who iteratively created a context specific curriculum based on feedback from Focus Group Discussions (FGDs) with local IMGs. Didactics, discussion, and role-plays were used to cover topics related to patient-centered care, challenging communication with patients, complex psychosocial histories, and health literacy. Post training surveys measured workshop satisfaction, levels of knowledge and skills related to patient-centered care and communication with patients and measured on the job performance of IMGs.

The use of theory to inform development of acculturation interventions is limited. The curriculum in the case study (17) was developed using the Kern’s curriculum development framework, an accepted six-step framework routinely used in practice to develop curricula for training of all physicians in different specialties (26). A conceptual framework about cross-cultural differences was utilized by Myers to guide curriculum development and analysis (20). Other trainings were developed based on the experiential learning of trainers of IMGs, personal experiences of IMGs who served as trainers, prior formal needs assessments or published research regarding IMGs needs. One program (18) was modeled on a pilot initiative developed for training of IMGs in family medicine at Canada’s McMaster University, but has since been discontinued (personal communication, dated 8/28/2021). The US had an induction training for IMGs, but it has been discontinued (personal communication from course creator). Recent developments (2025) at the American Medical Association have seen reconstitution of a committee to explore the strategies effective at acculturation of foreign-born IMGs (personal communication from an elected committee member).

Other one off, but relevant findings are that trainers of IMGs need to be aware of, and receptive to, the needs of IMGs (14) and ongoing training of existing staff creates collegial organizations more receptive to IMGs (15). Hosting IMG specific events (8). Acculturation training held specifically for IMGs as a one-day IMG specific event at the annual meeting of the American Psychiatrists Association allowed them to share their experiences and contribute to strategies to address the challenges they face and leverage opportunities for themselves and their families (18) till it was discontinued (4) (personal communication).

Only four studies (14, 16, 19, 20) in this group listed the 22+ home countries of their trainee.

### 3.2 Group II: studies that document IMGs’ perspectives on acculturation strategies they perceived to be helpful

They include qualitative studies (*n* = 17) (26–44) and a quantitative study (*n* = 1) (45). One study utilizes the grounded theory framework (34). Sample size of the qualitative studies varies from 8 (26) to 57 (42). The quantitative study (41) has a sample size of 371. One study (37) describes the creation of an IMG support group which moved from being an in-person group to the online mode during the COVID-19 pandemic. Fourteen of the 18 studies state the home countries of their respondents.

Studies regarding the perception of IMGs about strategies facilitating their transition to professional practice in their host countries (26–46) provide evidence that corroborates with level II evidence listed in the previous group. The major themes emerging from this group of studies is the perceived increase in self-efficacy of IMGs through induction training, collegial colleagues, a positive organizational climate, buddying with senior IMGs, shadowing physicians, clinical rotations, IMG support groups and caring communities. IMGs have expressed the need for tailoring the empowerment strategies to suit their unique needs (29, 35, 36). Only one (27) of the studies in this group had a curriculum based on a conceptual framework.

The main findings of this and the next group of studies are summarized at Table 5.

**Table 5:** Main findings of studies of perceptions of IMGs and expert opinions/viewpoints.

Author and year	Main findings
Studies of perceptions of IMGs	
Curran et al., 2008 (27)	Orientation training for IMGs is needed. It must be attentive to both professional and personal needs and should be comprehensive, multifaceted and sustained with inclusion of: details of the health care system and the peculiarities of the specific practice context in which they will be working opportunities for reflecting on one’s own cultural biases learning about the cultural background and beliefs of a new patient population. Mentoring and opportunities for effective integration within the community are also essential
Han and Humphreys, 2005 (28)	IMGs do not expect excessive support from the communities they practice in, but appreciate a welcoming culture and a supportive environment within the clinic and community they practice in. They expect tolerance, for they maintain their respective cultural and religious values and links with their respective ethnic communities.
Hashim, 2017 (29)	IMGs benefit from: mandatory induction program organized as teaching sessions on appreciation of the values and structure of the National Health Service (NHS), ethical and medico legal issues and different learning strategies in the UK receive feedback from colleagues supervised shadowing period prior in the first job in the UK assessment areas to be incorporated into the prequalifying examinations on NHS structure and hospital policies buddying schemes with senior IMGs educating NHS staff on different needs of IMGs
Hawken, 2005 (30)	Satisfaction with living in Finland, good team climate at work, cross cultural empathy, flexibility, patience, skill discretion and language skills essential for integration of Foreign-born physicians
Heponiemi, 2019 (46)	Facilitators: Financial and social support on arrival, through transition Support with language Own qualities and motivation Guidance from counselors during clerkships Prior medical knowledge
Huijskens et al., 2010 (31)	IMGs face challenges in their host countries in: accents, culture-specific sayings and non-verbal cues difference in doctors’ status relative to patients and different expectations of the doctor–patient relationship differences in patient confidentiality Training in cross-cultural communication skills would be beneficial to new IMGs
Lockyer et al., 2007 (32)	Collegial support in host communities is important for IMGs to acculture well in their host communities. In addition to studying for the professional licensing exams, IMGs needed to learn about regulations and systems, patient expectations, new disease profiles, new medications, new diagnostic procedures, and managing the referral process. This learning was facilitated by interaction with colleagues; the Internet, personal digital assistants (PDAs), and computers; reading; and continuing medical education programs. Patients stimulated learning and were a resource for learning.
Maddock and Kelly, 2017 (33)	IMGs found accents, culture-specific sayings and non-verbal cues challenging. IMGs’ differences in doctors’ status relative to patients and staff, expectations of the doctor–patient relationship were challenges and, at times, demotivating. Training in cross-cultural communication skills would be helpful Significant differences existed in attitude to patient confidentiality in Ireland versus the country of origin.
Malau-Aduli et al., 2020 (34)	IMGs stated they benefit from: information before departure from their country of origin, improved website information, more support for bridging courses, more observer programs, an IMG liaison officer at hospitals, reduction of the difficulties associated with passing the Australian Medical Council examination, support for their families, and relaxation of the rules about when and where IMGs can practice medicine.
McGrath et al., 2009 (35)	IMGs perceive a major shift from the culture of their country of origin (paternalistic doctor-dominated communication system; standard practice to talk to the family and not the patient) to the very different health care culture of Australia (more educated and informed consumers who demand high levels of information and discussion). IMGs benefit by learning about patient-centered communication upon arrival, during integration and practice. IMGs perceived the need for education on patient-centered communication to facilitate their integration into the Australian health system.
McGrath et al., 2012 (36)	IMGs find it helpful to have access to study groups and bridging courses
McGrath et al., 2013 (37)	Professional identity and sense of belonging of IMGs is constructed as an amalgamation of different identities—ethnic, gender, professional status, being as “other.” IMG participants reported that these events helped them build connections and better navigate practical aspects of living in the United States. In particular, the events improved their sense of well-being and helped them better cope with stress and adapt more easily to the local culture.
Neiterman et al., 2015 (38)	Depending on the context, feelings of belonging to a professional group (Canadian or Swedish) are fluid, ephemeral and changing. More attention to be paid to the social context in which experiences of processes of being *othered* and feeling *belonging* are being constructed and interpreted by people themselves.
Odebunmi, 2021 (26)	Observational study. Short talks by IMG panelists on topics they wished they had known about at the beginning of residency, such as culture shock, adapting and thriving in residency, and imposter syndrome. Feedback from new residents indicated that this event helped their anxiety about starting their residencies. IMGs’ orientation programs are helpful when they address the following: communication skills and English proficiency, including issues such as eye contact gender related issues adapt to learning styles in Canada: Medical education in Canada is based on principles of adult self-directed learning, is problem-based, focuses on the development of critical thinking skills, and emphasizes collaboration and relative equality between teacher and learner. In contrast, medical education in other cultures has been described as hierarchical, subject oriented, lecture-focused, and teacher-centered adaptation to ethical norms lack of exposure to mental health conditions lack of comfort in working in a collaborative team-based model with other health professionals adopt a more egalitarian relationship between the patient and physician and including the patient as an active member of the team who can make his/her own decisions about care
Rao, 2012 (45)	Quantitative study shows perceived increase in self efficacy of trainees through induction training, importance of a positive organizational climate for acculturation. IMGs would like more supervised interviews and experience feedback as excessively negative and critical
Slowther, 2012 (39)	IMGs need: Faculty and peer mentors to help them adapt and progress successfully in their residencies. For future IMGs, mentors should not be involved in their formal evaluations and assessment processes but should rather be able to focus on creating an open mentoring relationship. IMGs are very willing to help other IMGs
Snelgrove, 2015 (40)	Different inputs are needed to reach the same output; concept of equity with learner-oriented training
Umberin et al., 2019 (41)	I-IMG and C-IMG participants perceived two major challenges: discrimination because of negative labeling as IMGs and difficulties navigating their initial residency months. C-IMGs listed a third challenge: frustration around the focus on I-IMGs. Participants from both groups expressed mentorship as a useful strategy for acculturation and stated their desire to help other IMGs.
Wawdhane, 2007 (42)	IMGs and physicians find Clinical Attachments (CAs) useful—IMGs for the experience they gain and physicians for the additional manpower they get. They found the induction training useful. A third of the IMGs had been assessed at the end of their induction training.
Wearne et al., 2019 (43)	IMGs find the induction training and ongoing support helps them battle prejudice, orients them about host country culture, improves language and communication skills, and aids development of a new professional identity. They prefer modular training rather than having it all at one go. Supportive supervisors aid this process. IMG trainers feel IMGs need extra time to transition successfully into professional practice than the physicians trained in their host countries.
Wong and Lohfeild, 2007 (44)	IMGs benefitted from support from designated faculty mentors for IMGs; the second was peer support from other IMGs in training; and the third was sufficient time spent in the training program.
Viewpoints/commentaries	
Broquet and Punwani, 2014 (55)	IMGs in HIC most often have had their basic medical training in high hierarchical cultures. They have anxieties in receiving and giving feedback, They need to be provided with a fostering learning culture that values feedback as an expected and important part of all learning, ensuring that all (learners and supervisors) are trained in feedback skills, and clear setting of expectations.
Farag and Olaogun, 2020 (56)	There is a need for a national-scale effort to organize evidence-based teaching programs tailored to IMGs’ needs, using social media, for these have a wide reach among the target population of IMGs, and are easily accessible from all over the world.
Hamoda et al., 2014 (57)	Observership programs provide a unique opportunity to integrate IMGs into the U.S. Medical system. Benefits of observership programs to IMGs, psychiatry departments, and the U.S. Medical system as a whole are listed. A framework for establishing such programs in a way that will optimize their benefits and avoid potential pitfalls is provided. The different components of an observership program, core competencies that need to be acquired, challenges that observerships programs may encounter as well as recommendations for overcoming them are presented.
Jalal et al., 2019 (24)	Making the necessary information available in advance; create programs to bridge the cultural gap; have a “point of contact” responsible officer to be available to provide answers to questions and provide reference to available resources; have a national induction program.
Kehoe et al., 2018 (53)	A Culture of Support, individual needs assessment and access to—an adequate induction program, continuing support, supervision and a buddy are essential elements needed to acculturate IMGs to their host country. Action at the individual level should include job expectations, help develop a personal development plan and create a work-based assessment. Provision of detailed feedback increases confidence and self-awareness. Simultaneous action at the organizational level to increase staff cultural awareness and facilitate the development of a supportive culture, identify a champion, establish links within and between groups of IMGs and regional bodies.
Lagunes-Cordoba et al., 2021 (58)	Regulatory bodies to host IMG specific resources and disseminate them on a regular basis; professional associations of specialties to explicitly investigate differential attainment in the qualifying and licensing examinations; explicitly include IMGs in examination and curriculum design; publish data on IMG representation in these organizations; have IMG-specific events, resources and examples of best practice. Organizations: to have an IMG champion working with Human Resources to inform on IMG appointments; existing staff to have training to help them become competent to deal with IMG issues; explicit racism policy in place, displayed and implemented; local induction program, mainly during first 2 years of practice; focused support and mentoring of training; encourage and facilitate interaction of IMGs with graduates of host country; host events dedicated to their IMGs – to celebrate successful journeys and foster a sense of community; continuing professional development events to learn how IMGs’ experience in their host countries and how they can contribute positively to improved patient care. Individual level: pair IMGs with mentors; include modules focused on IMG issues for educational and clinical supervisors; encourage IMGs to attend local Balint groups; encourage IMGs to attend local academic days for trainees in specific specialties.
Ong, McFadden, and Gayen, 2005 (54)	Central induction course, complementing local trust-based induction programs, was developed for IMGs and evaluated by the London Deanery. Most participants found the course helpful, and their comments were used to further improve it.
Rao and Roberts, 2020 (5)	First comprehensive title on training IMG physicians in psychiatry. Developed by distinguished panel of US and IMG educators who have had deep experience in training IMGs. Focuses on the principles, practices and core clinical competencies that contribute to the making of a competent and ethical psychiatrist. Part I emphasizes how to understand the processes and components of psychiatry training and how to adapt to the local medical, social, geographical, ethnic, and religious cultures within the diversity US offers. This is highlighted for psychiatry is a discipline where nuances of language and culture clearly impact the delivery of care. It explains how programs presently operate, and the fundamentals of Graduate Medical Education, development of Core Competencies and Milestones, expectations that ACGME Accredited programs have for the graduation of competent psychiatrists. In addition, topics covered include the Doctor-Patient Relationship, the Psychiatric Interview, the Biopsychosocial Formulation, Psychotherapy, Professionalism and Ethics, and legal issues. Part II of the book provides an overarching description of the impact of immigration and identity development for the IMG and how to facilitate their transition and amalgamation in the US system. It also focusses on the issues Program Directors need to understand, such as visa issues, and provides guidance on how to prepare faculty members to appropriately supervise and give feedback to IMGs during the course of training.
Woodward-Kron, Fraser, Pill and Flyn, 2014 (47)	Evidence based Website developed (still functional)—functions as resource for IMGs for language and communication skills development. Doctors Speak Up http://doctorsspeakup.com/ is an open access English language resource for IMG doctors from non-English speaking backgrounds and their supervisors. It focusses on improving language and communication skills. It features video scenarios, which feature IMG doctors interviewing simulated patients. These serve as triggers for discussion in face-to-face teaching; has communication tasks highlighting effective, patient-centered communication skills. IMG doctors get to practice the language tasks in self-study mode while supervisors gain enhanced awareness of aspects of English that pose problems for IMGs from non-English speaking backgrounds. Over 19,500 users visited the website between March 2012 and November 2013.
Zaidi, Dewan and Norcini, 2020 (51)	There are opportunities for facilitation of IMGs’ transitioning to professional practice and enhancement of their careers at the time of their selection; pre-entry to their host country, entry, through training, start of practice career and during practice. Selection: Immigration reform to address visa issues IMGs face; residency spots to be increased nationally to address physician shortages; World Federation for Medical Education data to use quality standards to recognize accrediting agencies; provide space on national forums to highlight problems IMGs face; make available optional formal certificate programs for IMGs Pre-entry: Familiarize IMGs with nuanced U.S. professional behaviors, ethics, communications, and interactions through assignments using simulated doctor/patient video vignettes; orient IMGs to milestones and performance evaluation tools used during residency training Entry: Cultural orientation and assistance with day-to-day issues (in addition to standard institutional orientation process); IMG support group; Peer support group; Implicit bias training for faculty, staff, and trainees; Orientation to evidence-based medicine tools, responsible conduct of research, teamwork, and feedback skills Training and support: IMG support group; peer support group; mentorship; availability of well-being program and mental health support fostering IMG identity formation by preventing alienation from country of origin, denigration, and by providing environment where differences can help with transformational learning; recognizing specific challenges that women IMGs face and providing them with support. Start of practice career: Residency program directors to understand IMG visas and waiver programs; sessions for IMGs on negotiation skills and understanding contracts; incentivize practice in underserved areas During practice: Encourage IMGs to volunteer in country of origin or global medicine program; (if of interest); create IMG regional and national forums led by them and connected to national accreditation bodies; build national leadership programs for IMGs; institute national awards for IMGs for outstanding contribution to their field.

### 3.3 Group III: commentaries/perspectives/viewpoints/books

There is a recent spurt in the evidence using the lens of experiential learning to describe the IMGs trainers’ perspectives on “what works” to facilitate the acculturation of their trainees, for six (30, 32–34, 36, 38) of the 10 (29–38) papers described in this review were published since 2018.

Many trainers of IMGs are IMGs themselves and have the advantage of experiential learning of strategies effective in overcoming the challenges the new IMGs face (5). Observership programs in the US (30) are perceived to be beneficial to IMGs by Program Directors, in line with the finding about the usefulness of Clinical Attachments (CA) and shadowing by IMGs in the UK (42).

A different but related study documents the use of a website, “Doctors Speak Up” which functions as a resource for IMGs for language and communication skills development (47). The website can be accessed by IMGs before leaving their home country, in line with the recommendation information be provided to IMGs in advance (24). Although formal evaluation of this initiative is lacking, the fact that over 19,500 users visited the website between March 2012 and November 2013, and the website, now maintained by the University of Melbourne, continues to function till date, evinces that IMGs do find this website useful.

To summarize, findings of all studies included in this review, are listed, study-wise, at Table 6. Details of each study are available as Supplementary Table 1.

**Table 6:** Aggregation of findings, study wise.

Finding	Evidence
More information about host country before departure from home country; as in on professional body websites in host country for IMGs/ use social media; Access to study groups and bridging courses	Farag and Olaogun, 2020 (56); Jalal et al., 2019 (24); Lagunes-Cordoba et al., 2021 (58); Malau-Adali et al., 2020 (34); McGrath et al., 2009 (35); Woodward-Kron, Fraser, Pill and Flyn, 2014 (47); Zaidi, Dewan and Norcini, 2020 (51)
Induction training beneficial	Baker and Robson, 2012 (19); Bansal et al., 2015 (10); Bogle et al., 2020 (9); Cross and Smalldridge, 2011 (12); Curran et al., 2008 (28); Fry and Mumford, 2011 (13); Hashim et al., 2017 (29); Huijskens et al., 2010 (31); Jalal et al., 2019 (24); Katz et al., 2020 (14); Kehoe et al., 2018 (53), 2019 (8); Lagunes-Cordoba et al., 2021 (58); Malau-Ali et al., 2020 (34); McGrath et al., 2009 (35); Ong, McFadden, and Gayen, 2002 (54); Wearne, 2019 (42); Whyche, 2009 (18); Wright et al., 2012 (21); Zaidi, Dewan and Norcini, 2020 (51)
Mentoring opportunities beneficial	Bogle et al., 2020 (9); Curran et al., 2008 (28), Fournier et al., 2020 (11); Lagunes-Cordoba et al., 2021 (58); Slowther 2012 (39); Umberin et al.; 2019 (41); Zaidi, Dewan and Norcini, 2020 (51)
Welcoming culture and supportive colleagues, organization and community; point of contact/champion for IMGs; explicit racism policy in place—displayed and implemented	Bogle et al., 2020 (9); Broquet and Punwani, 2014 (55); Jalal et al., 2019 (24); Han and Humphreys, 2005 (29); Hawken, 2005 (30); Kehoe et al., 2018 (53); Lagunes-Cordoba et al., 2021 (58); Lockyer et al., 2007 (32); Rao and Roberts, 2020 (45); Zaidi, Dewan and Norcini, 2020 (51)
Training of organizational staff including training of trainers on different needs of IMGs to develop cross-cultural empathy	Bogle et al., 2020 (9); Hashim et al., 2017 (29); Hawkens, 2005 (30); Lagunes-Cordoba et al., 2021 (58); Zaidi, Dewan and Norcini, 2020 (51)
Supervised shadowing/Clinical Attachments /Observership program beneficial	Bogle et al., 2020 (9); Hamoda et al., 2014 (57); Hashim et al., 2017 (29); Wawdhane et al., 2007 (42)
Buddying schemes with senior IMGs	Hashim et al., 2017 (29); Kehoe et al., 2018 (8); Lagunes-Cordoba et al., 2021 (58); Slowther, 2012 (39); Zaidi, Dewan and Norcini, 2020 (51)
Observer program helpful	Hamoda et al., 2014 (57); Malau-Adali et al., 2020 (34)
Modular training—as add on to induction training	Baker and Robson, 2012 (19); Bogle et al., 2020 (9); Fournier et al., 2020 (11); Kehoe et al., 2019 (8); Wearne et al., 2019 (43)
Host IMG specific events for IMGs and to extend support to spouses and families	Broquet and Punwani, 2014 (55); Jalal et al., 2019 (24); Kehoe et al., 2018 (8); Lagunes-Cordoba et al., 2021 (58); Whyche et al., 2009 (18); Zaidi, Dewan and Norcini, 2020 (51)
Attention to be paid to the social context in which experiences of processes of being othered and feeling belonging are being constructed and interpreted by people themselves and there is a need for equity with learner-oriented training	Neiterman et al., 2015 (36); Snelgrove, 2015 (40)

## 4 Discussion

The studies included in this review are heterogenous in terms of the survey recruitment and administration methods, measurement instruments, types of interventions, timing of outcome measurements, and analytical methods, but their findings are complementary.

This review provides evidence that support for acculturation of IMGs can, and should, start before the foreign-born IMGs leave their home country. The form of support can include multiple formats, such as providing information about the host country and its health systems, giving access to study groups and bridging courses, and offering social support to get to know future co-workers and colleagues and gaining access to places to stay. The online channels of communication, social media and even the websites of professional bodies in host countries can serve as to connect IMGs with their prospective host countries.

Once the IMGs arrive, acculturation interventions start with the traditional induction training. Even this initial training is expanding in terms of content, types of trainers needed and delivery channels available. This is based on emerging stronger body of evidence, documenting objective measures of IMGs performance, health system comfort levels, and patient outcomes. Yet this work continues to remain constrained in scope, for only four studies included in this review provided objective evidence of effectiveness of interventions conducted and IMGs’ and their trainers’ positive perception of the intervention. More evidence is essential to identify the unique country-based strategies that need to be developed so that outcomes at the personal and professional level are optimal for IMGs, their patients and host country health systems.

Curricula should be based on evidence-based design frameworks (48). Only three (17, 20, 34) of the 46 studies included in this review used theory to inform the design of acculturation interventions, with the rest being based on experiential learning alone. This needs rectification for use of conceptual frameworks to create curriculum helps focus on key variables and leads to conclusions that are more generalizable (49). Theories of stress and coping strategies from clinical psychology may help explicate how to provide the support IMGs need to face the strain of being away from family and loved ones and the stress of being in new environments, especially under such challenging circumstances.

Only four of the 15 studies in Group I, but 14 of the 18 studies in Group II documented the many countries of origin of the IMGs. This perhaps reflects that when examined from the lens of the acculturation intervention, the unique identity of the IMGs does not bear prominence but comes center stage when examined from the perspective of the IMGs. Yet, even though the home countries were mentioned, none of the studies in this review made an attempt to offer acculturation strategy tailored to the needs of this diverse group of physicians, using the one size fits-all approach. Studies have made recommendations that the unique culturally specific needs and strengths of IMGs from 150 + countries be identified (37, 40, 50), enabling the tailoring of acculturation strategies to suit context specific needs (1) and this needs to be acted on.

Additional interventions need to include professional, personal and social support. At the professional level, opportunities such as observership and shadowing programs, clinical attachments, are helpful, as are additional modular training, mentoring opportunities, buddying schemes with senior IMGs, all delivered over an extended period of time. On the social and personal front, creating a welcoming and understanding atmosphere is essential. This can involve training of host country health system personnel, hosting IMG specific events and extending support to IMGs’ spouses and families.

This review highlights IMGs’ need for sustained long-term support, evidence for which is currently limited. IMGs will have differing perceptions and needs, based on their racial, ethnic and social backgrounds, iterating the varied importance they will inevitably place on balancing work, family and lifestyle, and their priorities will change as they move the varying work-, family- and age-related life stages.

While the initiatives documented strengthen the acculturation strategies available for IMGs, more needs to be done. Racism and “othering” based on micro-aggressions have been brought up to a small extent in the studies included in this review, with recommendations for point of contact/champion to be designated for IMGs and an explicit racism policy be in place, displayed and implemented. Gender issues have not emerged as a significant issue in the included studies. Yet, it is acknowledged that a significant percentage of the IMGs in the US come from LMICs (25, 51), where the cultures are hierarchical and significant gender-based disadvantage exists (25, 51). Political and economic uncertainties are other acknowledged factors affecting the migration and retention of IMGs (25). They may create an unfair climate for selection of IMGs for residency positions in the US (25) and limit the selection of the effect of bespoke support programs put in place to help IMGs navigate their professional careers in HICs. Action to rectify these factors requires action at levels outside the health system. Further, this review (1) recommends studies that document how variation in content and delivery of acculturation strategies would be of greater value than single intervention studies. Healthcare facilities vary considerably in terms of their structure, their organizational culture and the communities they serve and IMGs from different countries have diverse needs.

Finally, from a broad perspective, host country professionals may also benefit from targeted interventions to help create a more open and welcoming climate for professionals and patients from diverse races and ethnicities. This would help the country be better prepared for the future, for the US is projected to become a more diverse nation (52). At the time of publication, the American Medical Association has constituted a Committee to initiate training based on the unique needs of IMGs coming to the US.

## 5 Strengths and limitations

A strength of this study is the holistic search for information in all areas that can affect the intercultural doctor-patient relationship. The bibliography is related to education, addressing better communication, optimizing clinical performance, and cultural barriers in their performance.

There are a number of limitations is the inclusion of literature published in English. The sample sizes of the intervention studies are either not mentioned (53, 54) or are small, ranging from 5 (15) to 36 (14). Although efforts were made to include as many heterogenous studies as possible and compare the findings of published studies with unpublished research by contacting trainers of IMGs, publication bias may be a limitation. Among the intervention studies included in this review, none of the studies included a pretest in their outcome variable. Hence, it was not possible to rule out pre-intervention growth trajectory as an alternate explanation of the findings.

Another limitation may be origin of the literature – studies from North America, the UK, Australia would differ in their results based on the inherent differences in their health systems as well social structures. Also, the fact that studies consider all IMGs as a homogenous group itself is a limitation, the proportion of origin of the IMGs, for example, those from India vs. those from Latin American, and even the intra-group variation, as in IMGs from India, which constitutes the largest group of IMGs from a single country in many HICs, is large enough to require micro-tailoring of strategies for successful acculturation. Finally, strategies found successful in a health system may not be a good fit for another because of the variation in characteristics of health systems in different HICs.

## 6 Recommendations and conclusions

Creating and implementing acculturation strategies to help as transition to professional practice can positively impact their professional and personal lives and the patient care they deliver. Facilitating cross-country learning among HICs can optimally utilize resources and guide further research on variation in the content and delivery of acculturation strategies. There is scope for greater synergy between conceptual frameworks and practices to create more meaningful content and initiating a nuanced consideration of the diversity of IMGs and their experiences. There is need for more in-depth research to find evidence-based, context and racial and ethnic group-based strategies that are a good fit both, the diverse diaspora of IMGs and the individual country specific health systems. This would help health systems better respond to the evolving needs of an increasingly diverse HIC population, including that of the US.

## Data Availability

The original contributions presented in the study are included in the article/Supplementary material, further inquiries can be directed to the corresponding author.
