# Supplementary material for: Achieving equity for International Medical Graduates: a systematic review
Source: Front Med (Lausanne). 2025 Jul 23;12:1601492. doi: 10.3389/fmed.2025.1601492 (PMC12325190; doi:10.3389/fmed.2025.1601492)
Supplement: Supplementary file 1 [file Data_Sheet_1.pdf]

Appendix:

PubMed Ovid Search_SGS

3rd July 2021
Ovid MEDLINE(R) <1996 to June Week 4 2021>

1 International Medical graduate$.mp. [mp=title, abstract, original title, name of substance word, subject heading word, floating sub-heading word, keyword heading word, organism supplementary concept word, protocol supplementary concept word, rare disease supplementary concept word, unique identifier, synonyms] 769
2 limit 1 to (English language and yr="2000 -Current") 700
3 Foreign Medical Graduate$.mp. [mp=title, abstract, original title, name of substance word, subject heading word, floating sub-heading word, keyword heading word, organism supplementary concept word, protocol supplementary concept word, rare disease supplementary concept word, unique identifier, synonyms] 1898
4 limit 3 to (English language and yr="2000 -Current") 1538
5 Overseas trained doctor$.mp. [mp=title, abstract, original title, name of substance word, subject heading word, floating sub-heading word, keyword heading word, organism supplementary concept word, protocol supplementary concept word, rare disease supplementary concept word, unique identifier, synonyms] 36
6 limit 5 to (English language and yr="2000 -Current") 35
7 Overseas Trained Physician$.mp. [mp=title, abstract, original title, name of substance word, subject heading word, floating sub-heading word, keyword heading word, organism supplementary concept word, protocol supplementary concept word, rare disease supplementary concept word, unique identifier, synonyms] 0
8 limit 7 to (English language and yr="2000 -Current") 0
9 Foreign Trained Doctor$.mp. [mp=title, abstract, original title, name of substance word, subject heading word, floating sub-heading word, keyword heading word, organism supplementary concept word, protocol supplementary concept word, rare disease supplementary concept word, unique identifier, synonyms] 15
10 limit 9 to (English language and yr="2000 -Current") 13
11 Foreign Trained Physician$.mp. [mp=title, abstract, original title, name of substance word, subject heading word, floating sub-heading word, keyword heading word, organism supplementary concept word, protocol supplementary concept word, rare disease supplementary concept word, unique identifier, synonyms] 24
12 limit 11 to (English language and yr="2000 -Current") 22
13 2 or 4 or 6 or 8 or 10 or 12 1791
14 exp Social Discrimination/es, pc, px [Ethics, Prevention & Control, Psychology] 3068
15 limit 14 to (English language and yr="2000 -Current") 2993
16 Prejudice/ or Racism/ or Cultural Diversity/ 32338
17 limit 16 to (English language and yr="2000 -Current") 26784
18 Bias/ 20080
19 limit 18 to (English language and yr="2000 -Current") 16788
20 Prejudice/es, pc, px [Ethics, Prevention & Control, Psychology] 996
21 limit 20 to (English language and yr="2000 -Current") 974
22 Social Perception/ or Prejudice/ or Sexism/ 38869
23 limit 22 to (English language and yr="2000 -Current") 33314
24 15 or 17 or 19 or 21 or 23 61935
25 13 and 24 94
26 13 and 24 94
