# Supplementary material for: Achieving equity for International Medical Graduates: a systematic review
Source: Front Med (Lausanne). 2025 Jul 23;12:1601492. doi: 10.3389/fmed.2025.1601492 (PMC12325190; doi:10.3389/fmed.2025.1601492)
Supplement: Supplementary file 2 [file Table_1.pdf]

Supplementary Table 7

Study & Year	Sample size	IMGs home country	Host Country	Journal	Physician Specialty	Type of study	Intervention Duration	Intervention content	Pedagogy/Methods	Method of assessment	Categories of measures used*
**Intervention Studies**											
Baker & Robson, 2012[19]	14 IMGs	India, Pakistan, Sri Lanka, Libya, Sudan	UK	The Clinical Teacher	GPs	Case study	Training courses conducted by GP tutors and language tutors	Language & communication skills (for consultation)	Program run over 6 months, consisting of 15 sessions of language training delivered by a language tutor, and six sessions of consultation skills training from experienced GPs	GP tutors and language tutors (ongoing); supervisors (end evaluation through email questionnaire); IMGs(FGDs)	I,II,III,IV
Bansal et al, 2015[10]	7 IMGs	Not stated	UK	Education for Primary Care	Primary Medical Care	Case study	A full day course followed by a follow-up half day course two weeks later was piloted	One day course : morning – understanding patient centered approach (patient as a person; bio-psychosocial perspective; therapeutic alliance & sharing power & responsibility); afternoon – cultural diversity worksho	Videos, discussions, role plays, trainee led role plays, in class exercises;	IMGs: Oral feedback; documented; anonymous questionnaire Tutors: Meeting immediately after workshop	I

								p Follow up half day course: consultation skills			
Bogle et al, 2020[9]	Epsom Heller program, n=21 Kings Overseas Development Program n=not indicated	Not stated	UK	The Physician	Primary Medical Care	Case study	Epsom program: ;King's Overseas Doctors Development Program:	Epsom: 6 or 12 week orientation (pre-Covid)			III
Cross & Smalldridge, 2011[12]	20 participants per course; number of courses not specified	Not stated	UK	Medical Teacher	Primary Medical Care	Kings College: Leadership; NHS structure; ethical dilemmas, communication skills, interview practice, psychological support & well-being; regular teaching days(every 4-6 weeks)(Covid era) – different from UK	Epsom College: Clinical supervision, mentoring pastoral support Kings College: Faculty members trained in principles of coaching and mentoring; were IMGs themselves – used hi-fidelity simulatio	Epsom College: Multiple clinical assessments by supervisors Kings College: Feedback from IMGs; assessment by trainers at end of each training day			Epsom: I, III, Kings College: I

						Training Days	ns, FGDs, discussions, role plays, discussions, clinical scenarios, case presentations, face-to-face mentoring				
Fournier et al., 2020 [11]	17 mentor(peer volunteer IMG) & mentee(IMG) pairs	Not stated	Canada	Mental health and social inclusion.	Multiple	Case study	Two half day sessions followed by a one full day session	History taking, summarizing, discharge summaries, presentation skills and a communication model; cultural context	Presentations, workshops and practice using linguists, healthcare professionals and simulated patients	Language teachers – adjustment to syntax, pronunciation & context;<br>Clinicians – analyzed clinical content & structure required for verbal handover & written notes	II, III
Fry & Mumford, 2011[13]	13 IMGs who had previously been unable to clear the Clinical Skills Assessment Test; 6 passed after the	Not stated	UK	Education for Primary Care,	Primary Care	Case study	Short sessions conducted periodically over 2 years	Matching of mentors and mentees – frequency of contact, mode of discussion left flexible.	Peer mentoring. Session was offered for mentors to exchange, reflect and provide feedback on their experiences	Questionnaire using a five-point Likert scale assessing the perceived helpfulness of the program, satisfaction with the frequency and quality of contact with the mentor, and satisfaction with the mode of communication used (in-person, e-mails,	I

	intervention							Program administrators available to offer support/answer questions		text messages, etc.) developed separately for mentors and mentees – answered anonymously using Survey Monkey	
Katz et al., 2020[14]	36	China, India, Iraq, Egypt, Jamaica, Korea, Latvia, Nepal, Nigeria, Pakistan, Palestine, Syria, Taiwan	USA	MedEdPORTAL	Pediatrics	Case study	Three sessions: Session one: Three role plays of ten minutes each followed by discussion of unstated duration; Session two: case cards led discussion; Session three: focused examination for selected clinical condition	General principles of cross-cultural competency Language training	Behavior modelling/roleplay	Subjective feedback from trainees plus objective in the form of pass rate in Clinical Skills Assessment (CSA). Difficult to ascribe degree of improvement due to course versus individual study.	I,II,III
Kehoe et al, 2019[8]	mixed methods (10 data sets)	Not stated	UK	Report	Multiple	Case study	3 day workshop done for PG1 year residents annually over a	Four module curriculum developed covering topics	Didactics, discussion, and role-play	1-year follow-up survey reported the workshops resulted in IMGs':<br>1) improved understanding of US medical culture.	I,II,III,IV

							total period of 3 years	related to patient-centered care, challenging communication with patients, complex psychosocial histories, and health literacy.			
Makker et al., 2020[7]	Six NHS hospitals , n not stated	Not stated	UK	BMJ Simulation & Technology Enhanced Learning.	Primary Medical Care	Case study	2 days	Day one: Personal skills: concepts of mindfulness, being proactive, building resilience to deal with rejection and setbacks, building optimism, understanding the importance of robust well-being	Day one: interactive zoom meeting complemented by power point presentation<br>Day two: remote simulation with demonstration through pre-recorded scenarios<br>Debriefing by Zoom	Written feedback by educators and attendees	I

								strategies. Day two: Clinical skills			
Myers 2004[20]	12	Asian and Eastern Europe countries	USA	Academic Psychiatry	Psychiatry	Case study	Eight monthly, 1-hour seminars	Culture-specific information – individualist and collectivist; principles of cross-cultural competency; clinical psychiatry cases	Discussions; behavior modelling; presentations; role plays; guided independent study	Eight-item, Likert-type 7-point scale, post-then-pre-questionnaire	II
Pillai & Tran, 2019[15]	6	Not stated	UK	Future healthcare journal	Not stated	Case study	1 day	Four clinical scenarios: Protecting patient safety within the pressures of bed shortages; Decision on escalation care plans in a critically unwell patient without	High-fidelity simulation using manikin and simulated actors.	Questionnaire with a mixture of open and closed questions graded on a 6-point Likert scale used by attendees	I, III

								mental capacity; Management of needlestick injury and patient confidentiality in high-risk patients; Acting on a serious patient incident and communicating it to relatives as a duty of candor.			
Porter et al., 2008[16]	11	India (n=7) & others not mentioned	USA	Teaching and learning in Medicine	Internal Medicine	Case study	2 weeks pre-residency course	Information about US; general cross-cultural competency; medical knowledge and clinical skills	Discussions; behavior modelling; lectures; role modelling; presentations; simulation; mentoring; tutoring	Pre and post-test: 16-item test of medical knowledge and skills (instrument not formally validated)	I,II,III
Sockalingam et al,2015[17]	19	Not stated	Canada	Academic psychiatry	Psychiatry	Case study: prospective single site	1 day, case study	Evidence-based mental health (EBMH), psychiatr	Co-taught by faculty paired with psychiatry senior IMG trainees currently enrolled in the residency training program using role-plays, case-based	Curriculum evaluation consisted of participants completing questionnaires with a 5-point Likert scale to assess perceptions	I, II

								ic documentation, enhancing psychotherapy, learning communication challenges in psychiatry, learning and integrating feedback, and managing social isolation.	learning, demonstration (for EBMH resources),discussions, reflection, and lectures.	regarding the orientation day and their comfort with each of the topic areas covered in the curriculum at three time points: (1) pre-orientation, (2) immediately post-orientation, and (3) 3-month post-orientation.	
Whyche, 2009[18]	not stated	Not stated	USA	Psychiatric news.	Psychiatry	Case study	1 day IMG Institute at Annual Meeting of Psychiatrists	Supervision and feedback, cultural diversity in the U.S. health care system, and special issues of communication and the doctor-patient relationship in	Modeled after a program developed for IMGs training in family medicine at Canada's McMaster University. Lectures, informal discussions, video vignettes and small group discussions	Not stated	I,II

								psychiatry			
Wright et al., 2012[21]	n=15 IMGs completed the study	Sri Lanka, Philippines, Colombia, India, Bulgaria, Bangladesh, Iran, Afghanistan, Vietnam, China, Egypt, Bosnia	Australia	Rural and remote health	Not stated	Case study	offered in 5 sessions over 3 months.	Clinical knowledge, attitudes and skills; language, professional, cultural and communications skills	Simulation-based training. Training program was underpinned by a website offering diverse learning resources	Audio visual review of performance found positive directional changes for clinical and communication skills which remained sustained 3 months post course.	I,II, IV

**Study & Year**	**Sample size**	**IMGs home country**	**Host Country**	**Journal**	**Type of study**
Studies of perceptions of IMGs regarding what works					
Curran et al, 2008[23]	13 GPs/FM physicians, 6 specialists;4 administrators	Africa, South Asia & Middle East	Canada	Canadian journal of rural medicine. 2008;13:163-169.	Qualitative study interviews multicenter
Han & Humphreys., 2005[24]	57 Overseas Trained Doctors	Europe/ America, UK, Asia, Africa, Middle East	Australia	The Australian Journal of Rural Health	Qualitative study using life history perspective
Hashim, 2017[25]	17 questionnaire responses & 3 semi-structured	Majority of participants from India and Pakistan	UK	Advances in Medical Education & Practice	Qualitative study

	interviews of IMGs				
Hawken, 2005[26]	n=30	not stated	NZ	N Z Med J.	Qualitative (interviews)
Heponiemi, 2018[43]	n=371	Estonia, Russia, other EU countries , other countries	Finland	BMC health 11ervices research.	Quantitative
Huijskens et al., 2010[27]	N=32 IMGs	Middle East, South Asia	Netherla nds	Medical Education	Qualitative study using in-depth interviews
Lockyer et al.,2007[28]	N=19	South Africa; Pakistan, South America, United Kingdo m, Europe, and Japan.	Canada	Journal of Continuin g Education in the Health Profession s	Qualitative telephonic interviews
Maddock & Henderson, 2017[29]	n=16		Ireland	*European psychiatry*	Qualitative study using in-depth interviews
Malau-Aduli et al., 2020[30]	IMG(n=2 0) & supervisor s (n=5)		Australia	PLOS ONE	Qualitative approach employing grounded theory methods

McGrath et al., 2009[31]	n = 9	China, Yugoslavia (Bosnia), Philippines and Sri Lanka	Australia	Education for Health	Qualitative study using in-depth interviews
McGrath et al., 2012[32]	n = 10	China, Yugoslavia (Bosnia), Philippines and Sri Lanka	Australia	Australian Family Physician	Qualitative, iterative research methodology
McGrath et al., 2012[33]	n= 30	India ; Sri Lanka; Iran; South Africa; Sudan; Pakistan; Caribbean); Russia; Philippines; Indonesia; Egypt; Serbia; and Afghanistan	Australia	Australian health review.	Qualitative descriptive study using iterative, open-ended interviews
Neiterman et al., 2015[34]	n= 15 Swedish, 67 Canadian immigrant physicians	not stated	Sweden & Canada	Journal of the Royal Society of Medicine.	Qualitative, FGDs

Odebunmi, 2021[35]	not stated	not stated	USA(University of Minnesota)	International journal of medical education.	Qualitative observational study: IMG support group started in 2019 in University of Minnesota to help connect IMGs to support one another. Short-term goals are to continue to host social events and to work with the Graduate Medical Education office in updating an intern survival handbook
Rao, 2012[36]	N=62	Not stated	USA	Academic Psychiatry	Quantitative study
Slowther, 2012[37]	n=26	Pakistan; Nigeria ;India ; Italy; Greece; South Africa ;Hungary; Iran; Poland; Egypt; Russia; Spain; Germany; United States	UK	Advances in Health Sciences Education.	Qualitative study : interviews and FGDs
Snelgrove, 2015[38]	n=8	Itlay, Germany, Greece, Poland, Spain	UK	Medical teacher. 2019;41:1065-1072.	Qualitative studies : interviews & FGDs
Umberin et al., 2019[39]	11 IMGs (I-IMGs) and 11 Canadian IMGs (C-IMGs) Internal Medicine (9),	South Asia, Middle East, South America, and Eastern Europe	Canada	Advances in Health Sciences Education	Qualitative case study

	Family Medicine (7), and General Surgery (6)				
Wawdhane, 2007[40]	573 IMGs applying for a house officer post and 102 consultant physicians	South Asia, Middle East, Africa and European Economic Area (EEA)) and 102 consultants	UK	Postgraduate medical journal	Qualitative study through questionnaire for Opinion of IMGs and physicians about Clinical Attachments
Wearne et al, 2019[41]	16 expert informants from Australian group training stakeholder organizations; 12 international interviews from 10 international group training organizations in 5 countries – UK, Canada, Netherlands,	Australia, UK, Canada, Netherlands, Ireland, NZ	Australia	Medical Teacher	Qualitative interviews

	Ireland, NZ				
Wong et al, 2008[42]	n=12 IMGs		Canada	Medical Education	Qualitative interviews
**Authors**	**Country**	**Journal**			
Broquet & Punwani, 2014[44]	USA	Academic Psychiatry	Viewpoint/Commentary		
Farag & Olaogun, 2020[45]	UK	Cureus	Viewpoint/Commentary		
Hamoda et al., 2014[46]	USA	Academic Psychiatry	Viewpoint/Commentary		
Jalal et al., 2019[47]	UK	Future healthcare journal.	Viewpoint/Commentary		
Kehoe et al., 2018[48]	UK	The Clinical Teacher	Viewpoint/Commentary		
Lagunes-Cordoba et al., 2021[49]	UK	BJPsych Bulletin.	Viewpoint/Commentary		
Ong, McFadden, & Gayen, 2005[50]	UK	Hospital Medicine	Viewpoint/Commentary		
Rao & Roberts, 2020 [51]	USA	Book	Viewpoint/Commentary		
Woodward - Kron &		Medical teacher	Viewpoint/Commentary		

Robyn.2015 [52]			
Zaidi et al.,2020[53]	USA	Academic Medicine	Viewpoint/Commentary
